# Complete genome sequence of *Microbacterium* sp. strain Clip185

**DOI:** 10.1128/mra.01348-24

**Published:** 2025-02-25

**Authors:** Mautusi Mitra, Ana Stanescu

**Affiliations:** 1 School of Field Investigations and Experimental Sciences, University of West Georgia, Carrollton, Georgia, USA; 2 School of Computing, Analytics, and Modeling, University of West Georgia, Carrollton, Georgia, USA; Portland State University, Portland, Oregon, USA

**Keywords:** *Actinobacterium*, *Microbacterium*, Clip185, decaprenoxanthin, heavy metal tolerance, xenobiotic degradation

## Abstract

A xenobiotic-degrader and heavy metal-tolerant Microbacterium sp. strain Clip185 was isolated from a contaminated Tris-Acetate-Phosphate medium culture plate of a green micro-alga, *Chlamydomonas reinhardtii*. Here, we report the complete genome sequence of this strain to provide insights into its metabolic potential and nearest taxonomic neighbor.

## ANNOUNCEMENT

*Microbacterium* sp. strain Clip185 was isolated from a contaminated Tris-Acetate-Phosphate medium plate of *Chlamydomonas reinhardtii* strain, Chlamydomonas Library Project LMJ.RY0402.185141, obtained from the Chlamydomonas Resource Center as part of a local high school student science project at our lab (1). The genus was identified as *Microbacterium* sp. (MN633284.1, [1]) by partially amplifying and sequencing the 16S rRNA gene (1). As the strain is a heavy metal-tolerant, xenobiotic-degrading microbe (1), we sequenced its genome to gain insights into its survival strategies (1).

Genomic DNA was isolated from aerobically 28°C LB-grown cells using Qiagen’s Blood and Cell Culture DNA Mini Kit protocol without modifications. The genomic DNA sample was sequenced using PacBio single-molecule real-time (SMRT) sequencing. DNA was sheared using a Covaris g-TUBE. Post-shearing, genomic DNA fragments’ size range was determined with a Bioanalyzer 12000 chip, and DNA quantification was performed on a Nanodrop system. PacBio SMRTbell library preparation protocols as stated in the handbook (PacBio, CA, USA) were used to purify and concentrate 12kb fragment sizes, prepare SMRTbell library, and assess its quality and barcode. PacBio SMRT continuous long-read sequencing on a PacBio Sequel II System using PacBio Sequencing Primer v.4 was used to sequence the barcoded SMRT bell library. Reads were assembled and circularized using the SMRT Link version 9 software tools with built-in HGAP v4:0 powered by the PacBio Cromwell workflow engine. The pipeline was run at default with a pre-specified, approximately estimated genome size of 3.5Mb. The genome was also assembled with FLYE v2.9.1 (2) and CANU v2.2 (3) for statistical confidence. We used CANU v2.2 to generate corrected reads. Circlator (v1.5.5, 4) was used to detect and trim the overlap to circularize the assembly from the corrected and trimmed reads and the assembly file. Local assembly in Circlator was generated using built-in SPAdes-3.15.4 (5) with a kmer length of 107bp. *Microbacterium* sp. RG1’s DnaA gene sequence (accession number: CP034121.1) was used to rotate the genome using built-in Pymummer-0.11.0-py3.9.egg in Circlator. All above-stated tools were run with default parameters, unless otherwise specified. PacBio sequencing and the assembled contig statistics are shown in Table 1.

**TABLE 1:** PacBio sequencing and assembled contig statistics

Total number of subreads	1,276,627
Total number of mapped subreads	1,210,295
Mapped subread length N50 (bp)	11,108
Contig length (bp)	3,305,635
Contig L50	1
Contig circularity	Yes
G + C content (%)	69.5
Contig coverage (x)	3,193.6
Contig N50 (bp)	3,305,635bp
Contig & GenBank accession no.	Contig1/Chromosome CP117996.1

The assembled genome was deposited in GenBank (accession number GCF_028743715.1). The genome was annotated using the NCBI Prokaryotic Genome Annotation Pipeline (PGAP, 6) (v6.7 revision 2024–07-18.build7555; https://doi.org/10.6084/m9.figshare.28063082.v5; 6, 7). NCBI’s CheckM Analysis Tool (v1.2.2) was used to assess genome quality, which showed a completeness of 99.29% and a contamination of 1.38% (8). The genome was reannotated using RASTtk v1.073 in the DOE Systems Biology Knowledgebase (KBase) with default parameters (https://doi.org/10.25982/193023.110/2479104) (9–11). GTDB-Tk-v1.7.0 in KBase with default parameters (11, 12) and PGAP’s built-in average nucleotide identity (ANI) tool identified the strain genus as *Microbacterium* sp. PGAP identified the best-match type strain as *Microbacterium binotii* (accession number GCA_039532115.1), with an ANI of 91.38% (6, 13), indicating that Clip185 is likely a new *Microbacterium* species. Phylogenetic analyses based on the 16S rRNA gene using the KBase Build Phylogenetic Tree (https://doi.org/10.25982/193023.110/2479104; 9) from MSA using FastTree2-v2.1.11 (14) application revealed *Microbacterium* sp. RG1 (accession number: CP034121.1) as the nearest neighbor (6).

## Data Availability

The whole-genome sequencing project, including the PacBio DNA methylation motifs, has been deposited at GenBank under the accession number GCF_028743715.1. The raw sequence reads have been deposited in the SRA under the accession number SRR23538270. KBase analysis is publicly available in the KBase static narrative (https://doi.org/10.25982/193023.110/2479104). The PacBio DNA methylation motifs have been submitted to REBASE (Ref # 35996).
